# Integrating collaborative robots in manufacturing, logistics, and agriculture: Expert perspectives on technical, safety, and human factors

**DOI:** 10.3389/frobt.2024.1342130

**Published:** 2024-12-02

**Authors:** Luca Pietrantoni, Marco Favilla, Federico Fraboni, Elvis Mazzoni, Sofia Morandini, Martina Benvenuti, Marco De Angelis

**Affiliations:** Department of Psychology, Alma Mater Studiorum - University of Bologna, Bologna, Italy

**Keywords:** collaborative robots, human-robot collaboration, industrial automation, safety perception, trust development, workforce transition

## Abstract

This study investigates the implementation of collaborative robots across three distinct industrial sectors: vehicle assembly, warehouse logistics, and agricultural operations. Through the SESTOSENSO project, an EU-funded initiative, we examined expert perspectives on human-robot collaboration using a mixed-methods approach. Data were collected from 31 technical experts across nine European countries through an online questionnaire combining qualitative assessments of specific use cases and quantitative measures of attitudes, trust, and safety perceptions. Expert opinions across the use cases emphasized three primary concerns: technical impacts of cobot adoption, social and ethical considerations, and safety issues in design and deployment. In vehicle assembly, experts stressed the importance of effective collaboration between cobots and exoskeletons to predict and prevent collisions. For logistics, they highlighted the need for adaptable systems capable of handling various object sizes while maintaining worker safety. In agricultural settings, experts emphasized the importance of developing inherently safe applications that can operate effectively on uneven terrain while reducing workers’ physical strain. Results reveal sector-specific challenges and opportunities: vehicle assembly operations require sophisticated sensor systems for cobot-exoskeleton integration; warehouse logistics demand advanced control systems for large object handling; and agricultural applications need robust navigation systems for uneven terrain. Quantitative findings indicate generally positive attitudes toward cobots, particularly regarding societal benefits, moderate to high levels of trust in cobot capabilities and favorable safety perceptions. The study highlights three key implications: (1) the need for comprehensive safety protocols tailored to each sector’s unique requirements, (2) the importance of user-friendly interfaces and intuitive programming methods for successful cobot integration, and (3) the necessity of addressing workforce transition and skill development concerns. These findings contribute to our understanding of human-robot collaboration in industrial settings and provide practical guidance for organizations implementing collaborative robotics while considering both technological advancement and human-centered design principles.

## 1 Introduction

Recent research on collaborative robots (cobots) highlights their increasing adoption across various sectors, including automotive, logistics, and agriculture, primarily aimed at enhancing efficiency, productivity, and worker safety. Cobots are designed to work alongside human operators, enhancing productivity and safety while addressing the challenges posed by traditional automation methods. The integration of cobots into these sectors is driven by advancements in technology, particularly in artificial intelligence (AI), machine learning, which facilitate more intuitive human-robot interactions and operational efficiencies (Malik and Bilberg, 2019).

In the manufacturing sector, cobots are increasingly utilized to optimize assembly lines and improve operational workflows. They assist workers with tasks that are ergonomically challenging, thereby reducing physical strain and enhancing overall workplace safety. Studies indicate that cobots can significantly improve productivity by automating repetitive tasks while allowing human workers to focus on more complex activities that require cognitive skills (Borboni et al., 2023; Kakade, 2023). Furthermore, the implementation of cobots in manufacturing environments has been shown to foster a collaborative atmosphere that enhances worker satisfaction and reduces turnover rates (Othman and Yang, 2022).

In logistics, cobots are transforming supply chain operations by automating material handling and inventory management tasks. Their ability to navigate dynamic environments and interact with human workers makes them invaluable in warehouses and distribution centers. Research indicates that cobots can streamline logistics processes, reduce errors, and enhance the speed of operations, particularly in last-mile delivery scenarios (Pessot et al., 2023). The integration of AI-driven cobots allows for predictive maintenance and real-time data analysis, which further optimizes logistics operations.

The agricultural sector is also witnessing a surge in the use of cobots, particularly for tasks such as harvesting, sorting, and planting. These robots not only increase efficiency but also address labor shortages in the agricultural workforce. Studies have shown that cobots can improve the precision of agricultural tasks, leading to better crop yields and reduced waste. The collaborative nature of these robots allows them to work closely with human farmers, enhancing productivity while ensuring safety in potentially hazardous environments (Rowan, 2022; Guruswamy et al., 2022).

Despite the numerous benefits of cobots, challenges remain regarding their widespread adoption. Issues related to safety, trust, and the potential displacement of human workers are significant concerns that need to be addressed (Adel, 2022; Raja Santhi and Muthuswamy, 2023). Research emphasizes the importance of developing robust safety standards and training programs to ensure that both workers and cobots can operate effectively and safely in shared environments (Guertler et al., 2023). Additionally, as the technology evolves, continuous vocational training will be essential to equip workers with the necessary skills to collaborate effectively with cobots.

Human factors and ergonomics serve as fundamental elements in human-robot collaboration (HRC), significantly influencing interaction effectiveness, acceptance, and overall success (Green et al., 2008; Simone et al., 2022). Contemporary research has expanded into cognitive domains, examining user experience (Gervasi et al., 2022), cognitive load (Kim, 2022), and social cognition (Henschel et al., 2020). This heightened attention to human-related aspects aligns with the emerging Industry 5.0 (I5.0) concept, which represents an evolution from Industry 4.0. This transformation emphasizes the seamless integration of advanced technologies with human-centric approaches, particularly focusing on resilience, human wellbeing, and sustainability (Trstenjak, et al., 2022). While I5.0 encompasses various sectors, its impact is particularly significant in manufacturing (Narkhede et al., 2023), logistics (Berkers et al., 2023), and agriculture (Henriksen et al., 2022). These sectors stand at the forefront of the I5.0 revolution, where HRC dynamics play a pivotal role. Nevertheless, despite HRC’s growing importance in these settings, substantial gaps remain in understanding the challenges associated with workers’ safety and skills development within these new collaborative environments. Additionally, there is limited comprehension of the requirements and specific standards necessary for such innovative transformation (Villani et al., 2018).

The main objective of this study is to investigate the collaborative dynamics between workers and cobots across three distinct industrial sectors: vehicle assembly operations (manufacturing), robotic handling in warehouses (logistics), and vineyard operations (agriculture). These sectors represent diverse applications of HRC, each presenting unique requirements and challenges (Shamshiri et al., 2018). Through a questionnaire-based qualitative approach targeting technical professionals in automation and robotics, this research aims to comprehensively examine experts’ perceptions regarding technical, ethical, and safety aspects of cobot deployment.

The study specifically focuses on understanding three key dimensions: attitudes toward collaborative robots (Koverola et al., 2022), trust in robotic systems (Charalambous et al., 2015), and safety perceptions (Arents et al., 2021). This qualitative methodology enables the exploration of nuanced experiences and perspectives that quantitative methods might not capture, including workers’ adaptation to technological changes, job security concerns, and expectations about system efficiency (Stapels and Eyssel, 2021). Furthermore, this approach allows for examining contextual factors such as cultural attitudes towards technology and task-specific considerations in cobot applications (Søraa et al., 2023), providing insights into how assembly line workers perceive their interactions with cobots, including safety considerations and job satisfaction impacts (Bhargava et al., 2021).

Conducting qualitative studies to gather perceptions of targeted technical professionals in automation and robotics is crucial for understanding the collaborative dynamics between workers and collaborative robots in specific use cases. Qualitative research allows for an in-depth exploration of how assembly line workers perceive their interactions with cobots, including their feelings of safety, job satisfaction, and the perceived impact on their roles (Bhargava et al., 2021) but also can provide insights into how workers adapt to these changes, their concerns about job security, and their expectations regarding the efficiency of robotic systems (Stapels and Eyssel, 2021).

## 2 Literature review

### 2.1 Collaborative robots in manufacturing

Recent research on collaborative robots in manufacturing and assembly lines has explored various dimensions of HRC, revealing insights into how these interactions can be improved (Ajoudani et al., 2018; Kumar et al., 2021).

One significant area of research is the impact of cobots on worker productivity and posture. Bouillet’s study demonstrated that the introduction of a cobot in collaborative tasks resulted in longer collaboration times and increased production output, suggesting that the proactive coordination of cobots can enhance hybrid collaboration and improve overall productivity Bouillet (2023). This finding underscores the need to consider how cobots can be designed to facilitate smoother interactions with human workers, thereby reducing physical strain and improving ergonomic outcomes. Moreover, the relationship between human-cobot interaction fluency and job performance has been a focal point in recent studies.

Paliga’s research indicated that fluent and well-coordinated cooperation between humans and cobots positively affects job performance and satisfaction, regardless of the workload (Paliga, 2023). This highlights the importance of designing cobots that can adapt to the dynamic nature of human work, ensuring that operators feel a sense of control and fulfillment in their tasks. Such insights are critical for fostering a collaborative environment that enhances both productivity and worker wellbeing. The qualitative assessment of cobot interactions with different demographics, such as senior workers, has also been explored.

Rossato et al. (2021) found that cobots can significantly enhance the efficiency of manufacturing systems while improving the quality of life for human operators. This is particularly relevant as the workforce ages and the need for ergonomic solutions becomes more pressing. Understanding how different worker profiles interact with cobots can inform the design of adaptive workstations that cater to diverse needs.

Safety remains a paramount concern in HRC. The Cobot And Robot Risk Assessment (CARRA) method developed by Stone et al. emphasizes the need for safety assessments that consider the unique dynamics of human-cobot interactions (Stone et al., 2021). This method aims to improve fluency in safe interactions, highlighting the necessity of integrating safety protocols into the design and operation of cobots. Furthermore, the psychological aspects of human-cobot interactions have gained attention, particularly regarding mental workload and emotional states. Pluchino’s study utilized eye-tracking and cardiac activity indices to assess senior workers’ mental workload during assembly tasks with cobots, emphasizing the need for a human-centric approach in designing collaborative systems (Pluchino et al., 2023).

Kumar and colleagues (2021) made significant contributions to the understanding of various HRC techniques, in the manufacturing processes, highlighting the potential for enhanced productivity and efficiency through effective human-robot interaction. They explored both one-way and two-way collaboration models, which are essential for understanding how humans and robots can work together effectively. This classification helps in identifying the specific needs and challenges associated with each type of collaboration, thereby providing a framework for future research and practical applications in the field (Inkulu et al., 2021). Kumar et al. also addressed the challenges faced in implementing HRC techniques, such as safety concerns, the need for effective communication between humans and robots, and the importance of designing user-friendly interfaces. The most recent research highlights the role of advanced technologies, such as artificial intelligence and machine learning, in facilitating more intuitive interactions between humans and robots, allowing for adaptive responses based on real-time feedback from the work environment. Kumar and colleagues emphasized that leveraging these technologies can lead to more efficient workflows and improved safety outcomes, as robots can learn from human actions and adjust their behavior accordingly (Othman and Yang, 2022).

### 2.2 Collaborative robots in logistics

Recent research on collaborative robots in logistics has highlighted their potential in enhancing operational efficiency, safety, and flexibility within supply chain processes. The integration of cobots into logistics operations is increasingly seen as a critical component of the broader trend towards Logistics 4.0, which leverages advanced technologies to optimize logistics activities.

One of the primary contributions is the exploration of safety mechanisms for cobots operating in logistics environments (Kiangala and Wang, 2022). proposed an experimental safety response mechanism that utilizes Q-learning algorithms and speech recognition to enhance the safety of autonomous moving robots in smart manufacturing settings. This research underscores the importance of developing robust safety protocols that ensure safe interactions between human workers and cobots, particularly in dynamic logistics environments where the risk of accidents can be heightened (Kiangala and Wang, 2022). Additionally, Rautiainen et al. (2022) emphasized the significance of multimodal interfaces for intuitive human-robot interaction, which is crucial for effective collaboration in logistics. Their findings suggest that enhancing communication between humans and cobots can improve operational outcomes, as workers are better equipped to interact with and manage robotic systems. This aligns with the growing recognition that successful HRC relies not only on the robots’ capabilities but also on the quality of interaction and the ease of use of control systems (Saenz et al., 2022).

Wei et al. (2022) highlighted the role of automated guided vehicles (AGVs) in warehouse systems, where multi-robot collaboration is optimized through advanced path planning and obstacle avoidance techniques. This research illustrates how collaborative approaches can enhance the efficiency of logistics operations by enabling multiple robots to work together seamlessly, thereby reducing operational bottlenecks and improving throughput.

Lambrechts et al. (2021) conducted a comprehensive investigation into the human factors influencing the implementation of cobots in order picking operations, a critical component of warehouse logistics. Their research identified several key human factors that impact the successful integration of cobots, including resistance to change, organizational culture, communication regarding changes, and leadership support. They found that resistance to change is often rooted in fear of job displacement and a lack of understanding of the benefits that cobots can bring to the workforce. Effective communication and leadership are essential to mitigate these concerns and foster a culture of acceptance and collaboration between human workers and robots. This highlights the importance of addressing psychological and organizational barriers when implementing new technologies in logistics. Berkers et al. (2023) further explored the implications of human factors in the context of logistics automation, emphasizing the need for a human-centered approach to the design and deployment of cobots. Their research underscores the significance of creating intuitive interfaces that facilitate seamless interaction between humans and robots. They argue that understanding the cognitive load and physical demands placed on workers is crucial for optimizing HRC systems. Their findings align with the broader trend in logistics research that advocates for integrating human-centered design principles in the development of automated systems.

### 2.3 Collaborative robots in agriculture

Collaborative robots have been applied to enhance productivity, sustainability, and efficiency in farming practices. One of the significant contributions to the field is the exploration of HRC techniques that leverage the strengths of both humans and robots. Yerebakan and Hu (2024) provides a comprehensive review of current research on HRC in agriculture, emphasizing its potential to design modern agricultural systems that capitalize on the unique capabilities of both parties. This review underscores the importance of integrating human expertise with robotic precision, particularly in tasks such as planting, harvesting, and pest control, where human cognitive skills can complement robotic efficiency.

The ability of robots to communicate and coordinate their actions is crucial for tasks that require high levels of precision and adaptability. The safety and ergonomics of HRC in agriculture have also been addressed. Tagarakis et al. (2021) explored the use of wearable sensors to monitor human activity in collaborative agricultural environments, emphasizing the need for safety measures when humans and robots operate in close proximity. This research highlights the importance of creating a safe working environment that minimizes risks associated with human-robot interaction.

More recently, Adamides and Edan (2023) conducted a comprehensive review of HRC strategies and approaches in the agricultural industry and proposed that HRC systems could function as transitional solutions toward full automation, effectively combining robotic capabilities with human skills to address current technological limitations and streamline system design. The study emphasizes the importance of adopting a mixed-methods approach to examine the multifaceted nature of human social aspects, including experiential knowledge, practical implementation, and cultural considerations. The authors advocate for broad stakeholder engagement, particularly technical experts, in addressing social dimensions during the deployment of robotic systems. This comprehensive strategy ensures a balanced understanding of HRC technical and social aspects, ultimately facilitating more effective cobot integration and acceptance in agricultural settings.

### 2.4 Human factors in cobot integration

The integration of collaborative robots (cobots) in industrial settings represents a significant advancement in HRC, yet it brings complex challenges regarding safety, trust, and human acceptance. While the technical capabilities of cobots continue to evolve, understanding the human factors that influence their successful implementation remains crucial (Faccio et al., 2023).

This study stems from the theoretical foundations of HRC through multiple lenses. By investigating attitudes, trust development, and safety perceptions in three distinct industrial contexts - vehicle assembly, warehouse logistics, and agricultural operations - this research aims to provide comprehensive insights into the dynamics of HRC. The study combines expert opinions with quantitative assessments to understand both the technical requirements and human factors essential for successful cobot integration. This approach acknowledges that while cobots offer significant potential for enhancing workplace efficiency and safety, their effectiveness ultimately depends on the careful consideration of human perceptions, trust dynamics, and safety requirements within specific industrial contexts.

#### 2.4.1 Foundational aspects

Understanding the foundational aspects of HRC requires examining multiple theoretical frameworks that explain how humans and robots interact and integrate in workplace settings. Mubin et al. (2013) establish three primary categories of robot roles during activities: tool, partner, and tutor. In industrial settings, particularly where cobots are equipped with AI systems, additional roles such as supervisor may emerge. These varying roles determine different types of collaboration and significantly impact trust and safety perceptions, largely due to the technical complexities of cobot operations that may not be immediately transparent to users.

The concept of “functional organs” (Benvenuti et al., 2020; Mazzoni and Benvenuti, 2023) provides a crucial framework for understanding the integration of technological artifacts and humans. This concept emphasizes how combined human-robot performance can exceed individual capabilities of either party. However, not all tools qualify as functional organs; only those deeply integrated into human practices, evolving through repeated use to become true extensions of human capability and operating without conscious control, achieve this status.

Building on this foundation, Human-robot “coefficiency” (Lagomarsino et al., 2022; 2023) offers another vital theoretical perspective. This concept suggests that during HRC, where humans and automated systems share common objectives, individuals view the interaction as a holistic unit, similar to human-human interactions. They select actions aimed at maximizing overall efficiency rather than focusing on individual components. The application of coefficiency principles proves crucial for skills development and can enhance workers’ trust and safety perceptions within manufacturing ecosystems. Recent research by Vianello et al. (2023) supports these theoretical frameworks through empirical evidence, showing how humans adapt to changing roles and control strategies of collaborating robots. Their study, focused on a sawing task, revealed preferences for energy-efficient modes and collaborative interactions, emphasizing the importance of understanding human responses to cobot behavior in fostering trust and positive attitudes.

#### 2.4.2 Attitudes and acceptance

Attitudes toward collaborative robots in organizations span a spectrum of positive and negative perceptions (Savela et al., 2022; Koverola et al., 2022). Edison et al. (2003) emphasize the distinction between personal and societal attitudes, noting that individual enthusiasm for technology doesn't necessarily correlate with positive views of its societal implications. As Koverola et al. (2022) observe, personal attitudes might involve simple enjoyment or discomfort with robot interaction, while societal concerns often center on broader issues like workforce displacement. Recent research by Kaur et al. (2023) provides valuable insights into worker perceptions, finding that robots offering as-needed assistance were viewed more favorably than fully interventional or standoff robots, particularly regarding autonomy and job security. This finding highlights the critical role of cobot deployment strategy in shaping worker attitudes.

#### 2.4.3 Trust development and dynamics

The development of trust in HRC presents unique challenges, as workers initially experience uncertainty regardless of prior robotic system experience (Groom and Nass, 2007). Multiple studies emphasize trust’s crucial role in successful human-robot engagement (Lee and See, 2004; Kopp et al., 2021; Maurtua et al., 2017), linking it to enhanced efficiency and productivity (Charalambous et al., 2015).

Hancock et al. (2021), Hancock et al. (2023) have extensively studied trust factors in human-robot interaction, identifying robot performance, anthropomorphism, and transparency as key predictors. Their recent work proposes an elaborate interpersonal trust model incorporating non-human entities. Atchley et al. (2023) introduce the “contagion effect” concept in trust, where initial system-wide trust can shift to component-specific trust based on individual robot performance. Recent innovations, such as integrating Large Language Models (Ye et al., 2023), demonstrate how enhanced communication interfaces can significantly increase trust levels in HRC, pointing toward future directions in cobot development and integration.

#### 2.4.4 Safety perceptions

Safety perception in HRC encompasses users’ risk assessment and comfort levels during interactions (Bartneck et al., 2009). This perception fulfills basic human needs (Ryan and Deci, 2000) and represents a state where individuals feel protected from physiological and psychological harm (Dyreborg et al., 2022). Arents et al. (2021) classify collaboration levels into three categories: coexistence, cooperation, and collaboration, each presenting unique safety challenges. Recent studies (Sahin and Savur, 2022) demonstrate how robot behavior changes significantly influence human safety perceptions during collaboration.

## 3 Methodology

### 3.1 The use cases

This study emerges from the SESTOSENSO project, an EU-funded initiative involving a consortium of European universities, research institutions, and private companies. The project’s primary objective is to develop advanced sensing technologies for robots to enhance HRC effectiveness and safety. We investigate three distinct industrial sectors where collaborative robots and assistive systems are implemented to improve worker safety and operational efficiency: manufacturing, logistics, and agriculture. Each sector presents unique technical, safety, and ethical challenges for HRC implementation.

The research examines three specific use cases that exemplify different aspects of human-robot collaboration:

#### 3.1.1 Manufacturing: COBOT-worker cooperative assembly

This use case focuses on vehicle assembly operations where workers perform tasks requiring diverse postures and varying workloads. Cobots assist workers by supporting heavy components (such as vehicle roofs) and managing tool logistics. The complexity of this environment is heightened by the simultaneous use of exoskeletons and cobots in confined spaces like vehicle cockpits. Key challenges include collision avoidance and optimizing worker movements. To address these challenges, the project develops AI control strategies and enhanced sensorization for both cobots and exoskeletons, ensuring safe and efficient three-way cooperation in this dynamic environment (Fournier et al., 2023; Razin and Feigh, 2023).

#### 3.1.2 Logistics: dual arm handling of large objects

Set within an online grocery fulfillment center, this use case explores bi-manual robotic manipulation of large, bulky objects. The system features a specialized robotic setup with sensorized skin for enhanced object-handling capabilities. Human workers primarily serve supervisory and collaborative roles, intervening only when the robotic system requires assistance or guidance with complex manipulation tasks. This setup represents a shift in traditional human-robot interaction paradigms, emphasizing cognitive rather than physical collaboration.

#### 3.1.3 Agriculture: collaborative mobile manipulators for harvesting

This use case addresses the challenges of grape harvesting in hillside vineyards, where workers traditionally face significant biomechanical stress from manual handling, awkward postures, and repetitive movements. The proposed solution integrates worker-worn exoskeletons with autonomous mobile manipulators that provide physical assistance. The system actively monitors human features and working conditions to optimize biomechanical load reduction, enhancing physical ergonomics and supporting efficient farming operations.

### 3.2 Selection of participants

An online questionnaire was designed to investigate the perceptions and attitudes of technical experts in the collaborative robotics domain towards HRC within three settings: manufacturing, logistics, and agriculture. The selection of participants was based on a strategic approach to ensure that the sample consisted of technical experts with relevant experience and knowledge in collaborative robotics. The participants were chosen based on their professional backgrounds, roles within their organizations, and experience working with or near cobots.

The recruitment strategy targeted individuals with technical job profiles within the manufacturing and automation sectors. This approach was chosen to ensure the participants had the technical expertise to provide valuable insights into adopting cobots in various industrial settings. Including participants with different roles within their organizations, such as Technical Field Managers, Technical Field Specialists, and experts in Human Resources (HR) or Health, Safety, and Environment (HSE), allowed us to provide a holistic overview of cobot adoption. Technical Field Managers and Specialists were selected for their hands-on experience and knowledge of the technical aspects of cobot implementation. At the same time, HR and HSE experts were included to offer insights into the social, ethical, and safety implications of cobot adoption in the workplace.

### 3.3 Measures

The questionnaire consisted of two main sections. The first section aimed to gather qualitative data by focusing on three specific use cases’ technical, ethical, and safety aspects. This approach sought to provide a richer understanding and capture diverse viewpoints on deploying cobots in these distinct industries. Participants were presented with detailed descriptions of each use case and asked to respond to open-ended questions regarding the potential technical and safety issues and the social and ethical implications of implementing cobots in these scenarios.

The second section of the questionnaire employed established psychometric scales to quantitatively assess relevant psychological factors, including attitudes towards robots, trust in their operations, and perceptions of safety during interactions. These scales were carefully selected based on their reliability, validity, and relevance to the study’s objectives. Participants were asked to rate their agreement with a series of statements using a Likert-type scale, providing a standardized measure of their perceptions and attitudes.

Three pairs of researchers analyzed the responses to the open-ended questions, ensuring a comprehensive and balanced data elaboration process. Each pair independently reviewed the responses within the context of the specific use case scenarios, identifying initial categories and themes. Through an iterative process of comparison and synthesis, the researchers refined these categories into central themes that emerged as pivotal to the study’s objectives. This collaborative approach helped to minimize individual biases and enhance the reliability of the qualitative findings.

By combining open-ended questions and validated psychometric scales, this study offers a comprehensive examination of the human factors’ issues surrounding human-robot collaboration, as perceived by technical experts. The mixed-methods approach allows for a deep understanding of the complex interplay between technical, social, and safety considerations, providing valuable insights into the challenges and opportunities associated with the deployment of cobots in three distinct settings. The questionnaire was distributed using the Qualtrics online platform, and data collection took place from March to June 2023.

#### 3.3.1 Qualitative measures: cobot adoption in the three use cases

Participants were presented with detailed descriptions of three distinct use cases involving collaborative robots in various industrial settings. Each use case highlighted the specific challenges, objectives, and potential benefits of implementing cobots in that context. After reviewing each use case, participants were asked to provide their insights and opinions by answering three open-ended questions designed to capture key aspects of cobot adoption:

##### 3.3.1.1 Technical issues

“*What are the key technical issues of cobot adoption in this particular use case?”.* This question aimed to elicit participants’ views on the critical technical factors, challenges, and opportunities associated with implementing cobots in the given scenario. Participants were encouraged to consider efficiency, productivity, flexibility, and innovation potential.

##### 3.3.1.2 Safety issues

*What safety-related issues warrant careful consideration in the design and deployment of cobots in this given use case*?” This question focused on identifying the critical safety aspects that should be prioritized when developing and implementing cobots in the specific use case. Participants were encouraged to consider factors such as collision avoidance, human-robot interaction protocols, fail-safe mechanisms, and the potential risks associated with the specific tasks and environments.

##### 3.3.1.3 Social and ethical implications

“*How could cobots facilitate or impede ethical and social considerations within this context?”.* This question sought to explore participants’ perspectives on the potential social implications of cobot adoption. Participants were asked to reflect on how cobots might influence factors such as job displacement, workforce diversity, skill requirements, and overall social acceptance of the technology.

The open-ended nature of these questions allowed participants to provide rich, qualitative responses based on their expertise and insights. The questions were designed to ensure a comprehensive understanding of the participants’ perspectives on cobot adoption in each use case.

#### 3.3.2 Quantitative measures: attitudes, trust, and safety perception

##### 3.3.2.1 Attitudes toward cobots

The General Attitudes Towards Robots Scale (GAToRS; Koverola et al., 2022) was used to assess participants’ attitudes toward collaborative robots (cobots). This 20-item scale comprises four distinct dimensions, each containing the five items: 1) Personal Level Positive (P+): Measures the level of comfort and enjoyment during interactions with cobots (e.g., “I would feel comfortable working with a cobot.”); 2) Personal Level Negative (P-): Assesses levels of unease and anxiety surrounding cobots (e.g., “I would be anxious about making mistakes while interacting with a cobot”); 3) Societal Level Positive (S+): Evaluates positive viewpoints about the societal benefits of cobots (e.g., “Cobots can enhance human capabilities and productivity.”); 4) Societal Level Negative (S-): Quantifies reservations and concerns about the broader societal impacts of cobots (e.g., “Overreliance on cobots may lead to a loss of human skills”).

Participants responded to each item using a 5-point Likert scale (1 = completely disagree; 5 = completely agree).

##### 3.3.2.2 Trust toward cobots

The Trust Perception Scale - HRI (Schaefer, 2016), a 14-item scale, was used to measure the multidimensional nature of trust in cobots. This scale assesses trust based on various parameters, such as functionality, maintenance requirements, performance expectations, and safety features. Example items include “Most cobots meet the user or operator’s expectations” and “I would feel comfortable assigning a cobot a critical task.” Participants responded to each item using a 5-point Likert scale (1 = completely disagree; 5 = completely agree).

##### 3.3.2.3 Perception of safety during human-cobot interactions

A four-item scale developed by Weiss et al. (2009), initially used to study novice users’ experiences with humanoid robots, was adapted to evaluate participants’ perceptions of safety during interactions with cobots. The scale covers four aspects of safety concerns: 1) Fear of causing harm to the cobot (e.g., “I fear to use cobots, as an error might harm the cobot”); 2) Fear of self-harm (e.g., “I hesitate to use cobots for fear of making errors that will harm me”); 3) Perception of safety in the interaction (e.g., “I feel safe when working with cobots”); 4) Overall safety perception (e.g., “I perceive cobots as safe”). These items provide a multidimensional view of perceived safety, assessing varying levels of fear, confidence, and overall safety perception. Participants responded to each item using a 5-point Likert scale (1 = completely disagree; 5 = completely agree).

### 3.4 Participants

The study initially involved 64 respondents who began the questionnaire. After screening and data validation, 31 participants, coming from the European Countries of the project’s partners (England, France, Greece, Italy, Latvia, Netherlands, Spain, Sweden, Switzerland) were included in the final analysis. The technical experts had an average age of 40.4 years (with a range from 26 to 58) and were predominantly male, with 24 males (77.4%) and seven females (22.6%). Participants were professionals actively engaged in various sectors. Specifically, 35.5% of the participants were from robotics and automation, 29.4% were involved in manufacturing, 16.1% in packaging, 9.7% in the automotive industry, and 9.3% in the chemistry and agrifood sector. Regarding their roles within their organizations, 13 participants (41.9%) were Technical Field Managers, 11 (35.5%) were Technical Field Specialists, and 7 (22.6%) were experts in either Human Resources (HR) or Health, Safety, and Environment (HSE) fields. In terms of their experience with collaborative robots (cobots), 14 respondents (45.2%) were currently actively engaged with cobots at the time of the study, while 17 respondents (54.8%) had experience working near cobots within the last 5 years.

## 4 Results

### 4.1 Experts’ opinions on the use cases

Table 1 summarizes the key insights from experts’ opinions on the technical impacts, social and ethical considerations, and safety issues related to adopting collaborative robots (cobots) in three distinct use cases: vehicle assembly operations, logistics, and vineyard harvesting.

**TABLE 1:** Experts’ opinions on the three use cases.

Technical impacts of cobot adoption	Social and ethical considerations	Enhancing safety in cobot Design and deployment
Use case “vehicle assembly operations”		
• Enabling effective collaboration between cobots to predict and prevent collisions • Improving workload management by enabling cobots to support heavy parts or handle component and tool selection for workers • Determining the range of movement based on the specific task • Utilizing sensors, cameras, and machine learning to enhance recognition of the work environment	• Facilitating tasks for operators, reducing the need for particular skills or physical conditions • Addressing limitations in the use of exoskeletons for workers with limited motor functions • Assessing socio-economic impacts of potential worker substitution by cobots • Ensuring cobots support rather than replace jobs, enhancing working conditions • Making cobots adaptable and user-friendly for all workers	• Designing cobot tools to ensure safety (e.g., avoiding sharp or non-reversible tools) • Real-time analysis of human movement to predict potential collision points • Adjusting cobot velocity to mitigate risk to humans • Considering ergonomics and testing forces exerted on the operator’s body • Minimizing risk through careful design, using minimal weights and speeds
Use Case “Logistics”		
• Facilitating the management of large loads, reducing accidents from incorrect weight assessments • Eliminating errors in picking products through electronic identification (e.g., barcode or RFID) • Redesigning physical work environments for safe cobot operation alongside humans • Flexibility, versatility, and sensitivity in handling food products, minimizing space requirements • Ensuring safety for human workers and preventing food contamination	• Assisting workers with physically demanding tasks, potentially affecting job numbers • Enabling humans to undertake more complex activities while cobots handle simpler tasks • Reducing physical labor and reshaping workforce dynamics • Potentially replacing low-paid jobs, impacting social dynamics • Helping people with disabilities but raising concerns over job losses due to automation	• Ensuring operators cannot enter the cobot’s work area during hazardous operations (e.g., using proximity sensors) • Addressing the absence of mature safety standards for close operator-cobot interactions • Considering nearby operator interference and implementing instant motor stop measures • Providing special safety equipment and intrinsically safe systems that activate in anomalies • Ensuring cobot reactivity to human touch and robust handling of objects to prevent accidents
Use Case “Vineyard”		
• Autonomous mobile manipulators can enhance precision and reduce harmful movements, allowing workers to carry more grapes with less effort and in less time • Implement visual technology like cameras to compensate for environmental irregularities (ground conditions, fruit shape, etc.) • Integration of soil conservation techniques, including precision seeding and minimal chemical impact, to prevent soil fatigue	• Improvement of worker’s quality of life, leading to higher productivity and a reduced risk of musculoskeletal diseases • Enhancing job access for individuals with physical disabilities • Importance of reliability and ease of use in wearable devices • Addressing cultural readiness among agrifood operators to adopt new technologies • Addressing exploitation issues often associated with manual, labor-intensive tasks like harvesting	• Developing inherently safe applications, prioritizing the ability of cobots to halt operations immediately in both typical and atypical risks • Considering worker mobility on uneven ground surfaces to ensure safety and efficiency (e.g., balancing, weight distribution, and movement considerations in steep slopes) • Emphasizing the redundancy of sensors and robust mechanical designs as critical safety measures

#### 4.1.1 Experts’ opinions on the use case about vehicle assembly operation

Experts reported on the technical impacts of cobot adoption in vehicle assembly operations, where workers perform tasks with diverse postures, workloads, and complexity, often requiring exoskeletons to reduce biomechanical load. They emphasized the importance of developing accurate kinematic models and control systems for specific tasks, determining the range of movement, and utilizing sensors, cameras, and machine learning to enhance recognition of the work environment. Cobots can assist by supporting heavy parts, such as the vehicle roof, picking components and tools for workers, and reducing human injuries due to repetitive loads. Enabling effective collaboration between exoskeletons and cobots is crucial to predicting and preventing collisions and improving workload management. One engineer in the automotive sector stated: “*Cobots can improve workload management by assisting workers in handling heavy vehicle components, such as the roof, or by efficiently selecting and delivering the necessary tools and parts to the workers, streamlining the assembly process”.* Other issues are related to developing user-friendly interfaces and intuitive programming methods to enable easy deployment and adaptation of cobots for different assembly tasks.

Regarding social and ethical considerations, experts highlighted that cobots could assist with lifting parts while humans/exoskeletons perform precision operations, facilitating tasks for operators and reducing the need for particular skills or physical conditions. They also noted the importance of addressing limitations in using exoskeletons for workers with limited motor functions and assessing the socio-economic impacts of potential worker substitution by cobots. Adopting cobots may lead to job displacement for some workers, particularly those involved in repetitive and physically demanding tasks. Nevertheless, it can also create new job opportunities in areas such as cobot programming, maintenance, and supervision. Cobots can help reduce workers’ physical strain and risk of injuries, improving their overall wellbeing and job satisfaction. One expert stated: “*In my opinion, the integration of cobots in the assembly process can greatly assist operators by reducing the physical demands and skill requirements for certain tasks, thereby creating a more inclusive and accessible work environment for employees with diverse abilities and backgrounds”.* However, there may be concerns about the long-term effects of working closely with machines and the potential for over-reliance on technology. Implementing cobots may require workers to acquire new skills and adapt to working alongside machines, which could be challenging for some individuals and require significant training and support.

Safety issues in cobot design and deployment were a major concern for experts. They stressed the need for real-time analysis of human movement to predict potential collision points, adjusting cobot velocity to mitigate risk to humans, and incorporating data from external equipment like exoskeletons to prevent collisions. Considering ergonomics and testing forces exerted on the operator’s body, minimizing risk through careful design using minimal weights and speeds and designing cobot tools to ensure safety (e.g., avoiding sharp or non-reversible tools) were also deemed essential. They also highlighted the need for comprehensive safety training and education for workers to ensure they understand and adhere to the safety protocols when working with cobots and exoskeletons.

#### 4.1.2 Experts’ opinions on the use case “robotic handling in the warehouse”

Experts highlighted various technical impacts of cobot adoption in the logistics use case. They emphasized the importance of adaptability to the working environment, efficient pick and pack activities, and flexibility in handling food products while minimizing space requirements. Cobots can assist with physically demanding tasks, increase operational safety, and eliminate errors in picking products through electronic identification. Redesigning physical work environments is necessary for safe cobot operation alongside humans. Cobots can also facilitate the management of large loads, reduce accidents from incorrect weight assessments, ensure safety for human workers, prevent food contamination, and reduce movement speed and costs associated with technical safety solutions.

One robotics engineer in the logistics sector stated: *“The sensing skin and control algorithms need to be robust enough to adapt to the varying shapes, sizes, and weights of the objects being handled, ensuring a secure grip and smooth manipulation. Other issues include optimizing the robotic system’s performance to maximize throughput and efficiency in the warehouse setting and developing intuitive interfaces for human workers to monitor and intervene when necessary”.*

Concerning social and ethical implications, adopting bi-manual robotic manipulation systems in warehouse settings can lead to significant job role changes for human workers. Cobots can reduce physical labor and reshape workforce dynamics, but there are concerns about potential job displacement due to automation. Introducing cobots may replace low-paid jobs often held by immigrant workers, impacting social dynamics. However, cobots can also enable humans to undertake more complex activities while they handle simpler tasks. While some jobs involving manual handling of large objects may be displaced, new roles may emerge in areas such as robot supervision, maintenance, and exception handling. One industry expert noted: “*The introduction of these robotic systems will shift the focus of human jobs towards more high-level decision-making and problem-solving tasks, requiring workers to develop new skills and adapt to working alongside advanced automation technologies”*. However, there may be concerns about the potential impact on employment levels and job security, particularly for lower-skilled workers. Implementing these robotic systems may require significant retraining and upskilling efforts to ensure workers can effectively transition to new roles and responsibilities.

Experts extensively discussed safety issues in cobot design and deployment. They emphasized the importance of ensuring operators cannot enter the cobot’s work area during hazardous operations and using proximity sensors to manage the space between cobots and operators. Redesigning workspaces with inputs from engineers, architects, and health professionals is crucial. Experts also highlighted the need to address the absence of mature safety standards for close operator-cobot interactions, consider nearby operator interference, and implement instant motor stop measures. Proper training and clear communication are essential to prevent safety incidents. Providing special safety equipment and intrinsically safe systems that activate anomalies, evaluating potential impact speeds and trajectories to prevent crushing and shearing incidents, and ensuring cobot reactivity to human touch and robust handling of objects is critical for avoiding accidents.

One expert said: “*Regarding this use case, The use of cobots for this application will facilitate the management of large loads. It will reduce the possible accidents this handling could cause if incorrect assessments were made regarding the object’s weight.”* Another expert states: “*From the point of view of safety, it must be ensured that in certain operations, the operator cannot enter the work area of the cobot. For example, when a large and heavy load is at a certain height. The presence of proximity sensors capable of detecting the distance of the cobot from the operator can, for example, be useful for decreasing the movement speed of the cobot”.*

#### 4.1.3 Experts’ opinions on the use case “cobots in the vineyard operations”

In the vineyard use case, experts discussed the technical impacts of cobot adoption. Autonomous mobile manipulators can enhance precision and reduce harmful movements, allowing workers to carry more grapes with less effort and in less time. The availability of management infrastructures, such as WIFI connections, is crucial to support cobot operations. Cobots can improve workers’ quality of life, leading to higher productivity and a reduced risk of musculoskeletal diseases. The assistance of cobots can significantly reduce workers’ physical effort. Participants reported that the collaborative system may increase efficiency and improve the quality of life for vineyard workers through reduced physical effort and enhanced carrying capacity. Factors such as reliability, ease of use, and consistent proximity of the cobot were emphasized to maximize benefits.

Experts also emphasized the importance of reliability and ease of use in wearable devices. The integration of soil conservation techniques, including precision seeding and minimal chemical impact, can help prevent soil fatigue. The implementation of visual technology like cameras can compensate for environmental irregularities, such as ground conditions and fruit shape. As one agricultural robotics expert pointed out, “*The autonomous mobile manipulator needs to be able to navigate through narrow, uneven terrains and adapt to changing weather conditions while precisely locating and assisting the human worker”*.

Regarding social and ethical considerations, experts noted that while cobots may initially be perceived as awkward, they can potentially transform industry practices, especially in harsh environments. Cobots can enhance job access for individuals with physical disabilities, elevating work from a social perspective to be more technical. They can also attract a younger workforce, but managing the impact on current workers is essential. Addressing cultural readiness among agrifood operators to adopt new technologies is crucial, focusing on balancing cost reduction with investments in life quality. Cobots can eliminate physical ability disparities among workers, thus democratizing the field. They can also help address exploitation issues often associated with manual, labor-intensive tasks like harvesting. An expert noted, “*The introduction of assistive technologies like exoskeletons and mobile manipulators could greatly improve the working conditions and overall wellbeing of agricultural workers, who often face significant physical demands and health risks*”. On the other hand, there may be concerns about the potential displacement of jobs, particularly for low-skilled workers who may not have the necessary training or skills to adapt to working with advanced technologies. Additionally, these collaborative systems may raise questions about the equitable distribution of benefits and the potential widening of the skill gap between workers who can effectively use these technologies and those who cannot.

Safety issues in cobot design and deployment were a significant concern for experts. They emphasized the importance of ensuring cobots comply with safety standards, such as UNI EN ISO 10218, to guarantee worker protection in all operational scenarios. Developing applications that are inherently safe and prioritizing the ability of cobots to halt operations immediately in both typical and atypical risks is crucial. Experts also stressed the need to consider worker balance and mobility on uneven ground surfaces to ensure safety and efficiency. Special attention should be given to balancing, weight distribution, and movement considerations, especially in areas with steep slopes, to prevent accidents. The redundancy of sensors and robust mechanical designs were highlighted as critical safety measures.

Regarding safety issues, participants stressed the importance of implementing reliable stability control and collision avoidance mechanisms to ensure the safe interaction between the human worker and the mobile manipulator, particularly on steep and uneven terrains. One safety engineer commented, “*The collaborative system should be designed to detect and respond to the human worker’s movements and potential loss of balance, adjusting its actions accordingly to minimize the risk of injury. We need to consider the steep surface of the ground. Moreover, the robotic system should not limit mobility”*.

In vineyard operations, unique challenges are posed by the uneven terrain and slope gradients characteristic of vineyards. Participants stressed that the design of cobots for vineyard operations should prioritize the stability and balance of both the cobot and the human worker. They suggested that cobots should be equipped with features that enable them to navigate rough terrain without compromising the safety or mobility of their human collaborators. Participants underscored the need for cobots to have high situational awareness and adaptability, by dynamically adjusting their movements and behavior based on real-time data, cobots can maintain a safe working distance from human workers and minimize the risk of accidents or injuries.

### 4.2 Attitudes towards collaborative robots

The General Attitudes Towards Robots Scale (GAToRS) was used to assess participants’ attitudes toward collaborative robots (cobots) across four dimensions: Personal Level Positive (P+), Personal Level Negative (P−), Societal Level Positive (S+), and Societal Level Negative (S−).

The mean scores for the P+ dimension ranged from 2.42 to 4.15, indicating a moderate to high level of positive attitudes towards cobots on a personal level. Participants expressed trust in the development of cobots (M = 4.15) and believed that the needs and feelings of users would be considered (M = 4.15). However, the lowest mean score in this dimension was for the item “If cobots had emotions, I would be able to befriend them” (M = 2.42), suggesting some hesitation in forming emotional connections with cobots.

The mean scores for the P- dimension were relatively low, ranging from 1.39 to 2.27, indicating that participants generally had low levels of negative attitudes towards cobots on a personal level. Participants expressed minimal fear (M = 1.39) and nervousness (M = 1.39) around cobots. The highest mean score in this dimension was for the item “I don't want a cobot to touch me” (M = 1.97), suggesting some discomfort with physical contact with cobots.

The mean scores for the S+ dimension were high, ranging from 3.91 to 4.38, indicating strong positive attitudes towards the societal benefits of cobots. Participants believed that cobots could make life easier (M = 4.38), allow people to do more meaningful tasks (M = 4.34), and help society by assisting people (M = 4.06).

The mean scores for the S- dimension were moderate, ranging from 2.13 to 3.72. Participants expressed some concerns about the societal impact of cobots, such as the need for close monitoring of robotics (M = 3.72) and the potential for societal upheavals due to unregulated use (M = 3.37). However, they were less concerned about cobots taking away jobs (M = 2.13) or encouraging less interaction between humans (M = 2.16).

**FIGURE 1:**
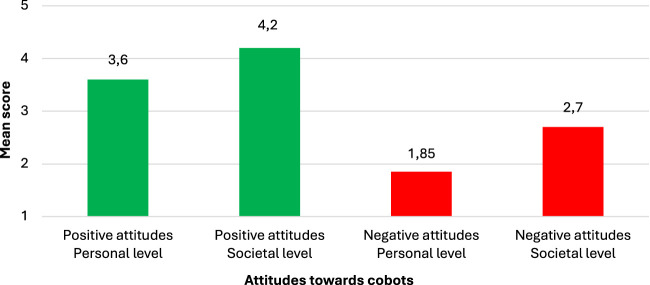
Attitudes towards cobots on personal and societal levels.

Overall, the results suggest that participants held generally positive attitudes towards cobots, particularly regarding their societal benefits, as shown in Figure 1. While there were some concerns about the societal implications of cobot adoption, personal-level attitudes were mostly positive, with low levels of fear and unease.

### 4.3 Trust in human-cobot interactions

The Trust Perception Scale-HRI was used to assess participants’ trust in collaborative robots (cobots) across 14 items. The means for these items ranged from 2.23 to 4.10, indicating a moderate to a high level of trust in cobots. The items with the highest means (above 3.70) suggest a strong belief in cobots’ ability to perform tasks successfully, follow instructions, and even outperform novice human users. Participants expressed high trust in cobots’ capability to do exactly as instructed (M = 4.10), succeed when performing tasks (M = 3.81), and be qualified for specific tasks (M = 3.81). Additionally, the reversed Item 13 (M = 3.77) indicates that participants believe cobots can perform tasks better than novice human users.

**FIGURE 2:**
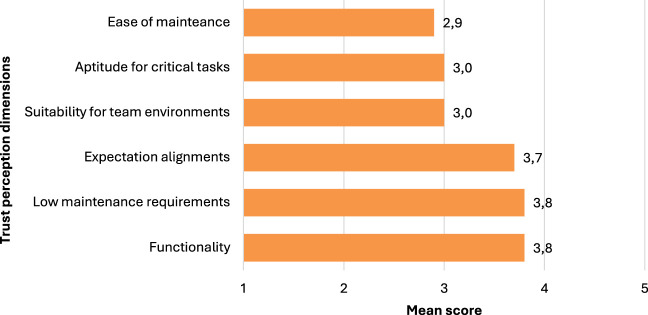
Trust towards cobots.

Items with moderately high means (between 3.30 and 3.70) indicate a reasonable level of trust in cobots’ ability to function in team environments, provide appropriate information, meet user expectations, and warn of potential risks. However, items with lower means (below 3.30) suggest relatively lower trust in cobots’ ability to be good teammates, work well in teams, and their maintenance requirements (see Figure 2).

### 4.4 Safety perception in human-cobot interaction

**FIGURE 3:**
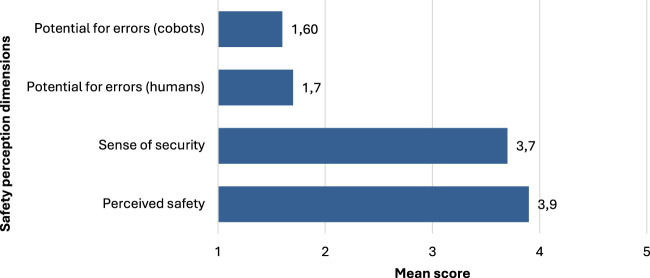
Safety perceptions of collaborative robots.

The evaluation of perceived safety in human-cobot interactions encompassed four specific items to evaluate the extent of apprehensions and participant confidence level. The results reveal a generally favorable perception of safety (Figure 3). Participants indicated high perceived safety (M = 3.9; SD = 1.0) and felt secure while working with cobots (M = 3.7; SD = 1.0). Conversely, concerns about potential errors that could harm the participants (M = 1.7; SD = .09) or the cobots (M = 1.6; SD = 0.8) were notably low.

## 5 Discussion

The present study aimed to investigate the perceptions and attitudes of technical experts towards adopting collaborative robots in three distinct use settings: vehicle assembly operations, robotic handling in warehouses, and agricultural harvesting. The findings provide insights into the technical, safety, and social implications of implementing cobots in these industrial settings.

Regarding the vehicle assembly use case, experts highlighted the importance of developing accurate and reliable sensor systems for seamless and safe interaction between workers, cobots, and exoskeletons. These findings align with previous research that underscores the significance of sensor technology and motion planning in human-robot collaboration (Robla-Gómez et al., 2017; Villani et al., 2018). Experts also stressed the need for user-friendly interfaces and intuitive programming methods to facilitate the easy deployment and adaptation of cobots for various assembly tasks, echoing the importance of usability in implementing industrial robots (Weiss and Spiel, 2022). Usability is also one of the most relevant factors in transforming a tool into the technological part of a functional organ, allowing humans to feel comfortable and trusted when using a specific tool (Mazzoni, 2017).

In the robotic handling in warehouses use case, experts emphasized the development of advanced sensing and control systems to ensure precise and reliable handling of large, bulky objects. Optimizing the robotic system’s performance to maximize throughout and efficiency was also highlighted, consistent with the goals of warehouse automation (Azadeh et al., 2019). Developing intuitive interfaces for human workers to monitor and intervene when necessary was also stressed, reinforcing the need for effective human-robot interaction in logistics settings (Rojas and Rauch, 2019). This aspect is also critical for the coefficiency of human-robot interaction, particularly in selecting actions aimed at maximizing the overall efficiency of the joint effort, achieving the best efficiency, and minimizing the probability of errors.

For the agricultural harvesting use case, experts identified developing robust navigation and localization systems as a key challenge for mobile manipulators operating in unstructured and dynamic vineyard environments (Kostavelis et al., 2017). This finding resonates with the current research focus on developing autonomous navigation systems for agricultural robots (Shamshiri et al., 2018). The importance of implementing reliable stability control and collision avoidance mechanisms to ensure safe interaction between human workers and mobile manipulators was also emphasized, particularly in steep and uneven terrains characteristic of vineyards. These are both critical for the evolution of a functional organ, allowing humans to overcome their limits and achieve better results and for the best effectiveness of the human-robot coefficiency.

Across all use cases, safety emerged as a paramount concern. Experts consistently highlighted the importance of implementing robust collision avoidance systems, fail-safe mechanisms, and emergency stop protocols to ensure the safety of human workers interacting with cobots. The emphasis on developing comprehensive safety protocols and the need for broad safety training and education for workers was also emphasized, underlining the crucial role of human factors in the successful adoption of cobots.

The social and ethical implications of cobot adoption were also explored. Experts recognized the potential for cobots to facilitate the inclusion of workers with diverse physical capabilities and limitations, promoting a more inclusive and accessible work environment. This finding aligns with the growing interest in using assistive technologies to support workers with disabilities in industrial settings (Bianchini et al., 2022). However, concerns about job displacement, particularly for workers involved in repetitive and physically demanding tasks, were also raised. This highlights the need for proactive measures to support workforce transitions and reskilling efforts (Li, 2022). Experts also noted the potential for cobots to reduce physical strain and risk of injuries for workers, improving overall wellbeing and job satisfaction. This finding is consistent with research demonstrating the ergonomic benefits of human-robot collaboration (Schmidtler et al., 2015). Addressing these concerns is urgent for formulating policies and creating organizational practices that guarantee the equitable allocation of benefits derived from the adoption of cobots. Scholars, organizational stakeholders, and policymakers are encouraged to leverage these insights to construct agendas that harmonize technological progress with social equity, ensuring that automation’s dividends are equitably distributed throughout society (Weidemann et al., 2023; Mazzoni and Benvenuti, 2015).

The quantitative measures employed in this study provide further insights into technical experts’ attitudes, trust, and perceptions of safety towards cobots. Participants exhibited generally positive attitudes towards cobots at both personal and societal levels, with higher positive attitudes at the societal level. This finding suggests that experts recognize the potential benefits of cobots for society, such as increased productivity (Gombolay et al., 2017). However, negative attitudes, particularly at the societal level, indicate that concerns about the broader impacts of cobot adoption, such as job displacement and skill gaps, persist and need to be addressed.

Trust perception in human-cobot interactions was found to be moderately high, with participants expressing confidence in the functionality, low maintenance requirements, and expectation alignment of cobots. This finding aligns with previous research highlighting the importance of trust in successfully implementing industrial robots (Charalambous et al., 2015). However, lower scores were observed for cobots’ suitability for team environments, aptitude for handling critical tasks, and ease of maintenance, suggesting areas for improvement in cobot design and integration.

Perceived safety in human-cobot interactions was generally favorable, with participants indicating high levels of perceived safety and security while working with cobots. The findings of this study contribute to the growing body of literature on human-robot collaboration in industrial settings. By providing insights into the perspectives of technical experts on cobot adoption in three distinct use cases, this research highlights the critical technical, safety, and social considerations that need to be addressed to ensure the successful implementation of collaborative robots. The results also underscore the importance of considering human factors, such as attitudes, trust, and safety perceptions, in the design and deployment of cobots.

This study has some limitations that should be acknowledged. The sample size may not represent the broader population of technical experts in the field. Additionally, our focus on the industrial, logistics, and agricultural sectors limited our ability to explore the unique challenges and requirements of other domains (Medical, HoReCa) where human-robot collaboration is equally important. Therefore, future research should aim to address these limitations by conducting larger-scale studies across various industries and contexts. Future research could also benefit from larger and more diverse samples to enhance the generalizability of the findings. Additionally, the study relied on self-reported data, which may be subject to response biases. Future studies could employ observational or experimental methods to triangulate the findings and provide a more comprehensive understanding of human-robot collaboration in industrial settings. Furthermore, ongoing research efforts should consider longitudinal studies that track changes in attitudes, trust, and safety perceptions as collaborative robots become smaller, more advanced, and widely adopted across different sectors. Continuously surveying the same areas of cobot deployment while expanding into new industries and application contexts will help assess how technological advancements and broader use cases influence the evolving dynamics of human-robot collaboration.

## 6 Conclusion

This study provides comprehensive insights into the implementation of collaborative robots across three distinct industrial sectors: vehicle assembly, warehouse logistics, and agricultural operations. Through the analysis of expert opinions and quantitative assessments of attitudes, trust, and safety perceptions, key findings emerge that have important implications for both theory and practice. The study’s examination of specific use cases reveals distinct challenges and opportunities. In vehicle assembly operations, the integration of cobots with exoskeletons presents unique challenges requiring sophisticated sensor systems and motion planning. For warehouse logistics, the emphasis lies in developing advanced control systems for handling large objects while maintaining human supervisor safety. In agricultural settings, the need for robust navigation systems and stability control on uneven terrain emerges as a critical consideration.

These findings have significant practical implications. Organizations implementing cobots should prioritize comprehensive safety training and user-friendly interfaces. System designers should focus on enhancing cobot capabilities in teamwork scenarios and maintenance accessibility, while industries need to develop proactive strategies to address workforce transitions and skill development. From a policy perspective, the findings underscore the need for standardized safety protocols across different industrial applications. They emphasize the importance of balancing technological advancement with workforce protection and highlight the requirement for guidelines that ensure equitable distribution of cobot-derived benefits.

In conclusion, while the implementation of cobots across different industrial sectors shows promise, success depends on carefully balancing technical capabilities with human factors. This study’s findings emphasize that effective cobot integration requires not only advanced technological solutions but also careful consideration of human perceptions, safety requirements, and societal implications. The insights gained from this research contribute to our understanding of how to effectively implement cobots in various industrial settings while maintaining focus on both technological advancement and human-centered considerations.

## Data Availability

The raw data supporting the conclusions of this article will be made available by the authors, without undue reservation.
